# Does postoperative chemotherapy improve overall survival of patients with ypT1-2N0 cancer?

**DOI:** 10.1186/s12957-022-02881-y

**Published:** 2022-12-27

**Authors:** Pengfei Su, Yingjing Zhang, Tian Yu, Lin Jiang, Weiming Kang, Yuqin Liu, Jianchun Yu

**Affiliations:** 1 Department of General Surgery, Peking Union Medical College Hospital, Chinese Academy of Medical Sciences and Peking Union Medical College, Beijing, 100730 China; 2 Graduate School, Chinese Academy of Medical Sciences and Peking Union Medical College, Beijing, 100005 China; 3 Department of Pathology, Institute of Basic Medical Sciences, Chinese Academy of Medical Sciences and Peking Union Medical College, Beijing, 100005 China

**Keywords:** Gastric cancer, ypTNM, Perioperative chemotherapy, Postoperative chemotherapy, Survival

## Abstract

**Background:**

Perioperative chemotherapy combined with curative gastrectomy has been increasingly represented the standard therapeutic strategy for resectable gastric cancer (GC). However, it is still unclear whether postoperative chemotherapy has a survival benefit for ypT1-2N0 gastric cancer patients who have undergone preoperative chemotherapy followed curative gastrectomy.

**Methods:**

The data of patients who undergone neoadjuvant chemotherapy followed by gastrectomy and had pathological classification of ypT1–2N0 between March 2016 and December 2020 at Peking Union Medical College Hospital were retrospectively reviewed. Chi-square test was adopted to compare the difference between the patients with postoperative chemotherapy (pCHT) and without postoperative chemotherapy (no pCHT). Survival curves for overall survival (OS) were estimated using the Kaplan-Meier method, and the log-rank test was used to compare survival difference. Univariate and multivariate analyses for prognostic factors were based on the Cox regression.

**Results:**

A total of 134 patients met the inclusion criteria and 56 (41.8%) of them have undergone postoperative chemotherapy. There were no statistically significant differences in demographic and clinicopathologic characteristics between pCHT group and no pCHT group (all *p* > 0.05). Postoperative chemotherapy was not associated with a significant improvement in overall survival (OS) (Hazard ratio [HR] 0.815, 95% confidence interval [CI] 0.403–1.650; *p* = 0.474). Subgroup analyses demonstrated survival was equivalent between pCHT and no CHT group in ypT1N0 patients (HR 0.832, CI 0.222–3.121; *p* = 0.786) and ypT2N0 patients (HR 1.284, CI 0.564–2.924; *p* = 0.551). Multivariable analysis identified that clinical T stage independently influenced prognosis (cT3 vs. cT2: HR 2.875, 95% CI 0.998–8.281, *p* = 0.050; cT4 vs. cT2: HR 7.382, 95% CI 2.569–21.211, *p* < 0.001). In clinical T3–4 patients, there was an overall survival benefit for postoperative chemotherapy (HR 0.270, 95% CI 0.114–0.634; *p* = 0.006). No survival benefit of postoperative chemotherapy was identified in clinical T2 patients (HR 0.689, 95% CI 0.200–2.372; *p* = 0.579). Furthermore, postoperative chemotherapy was proved to be an independently positive prognostic factor for clinical T3–4 patients (HR 0.132, 95% CI 0.051–0.345; *p* < 0.001).

**Conclusion:**

Postoperative chemotherapy might offer survival benefit to patients with ypT1-2N0 gastric cancer whose clinical T stage was T3–4 before preoperative chemotherapy.

## Introduction

As an aggressive malignancy with poor prognosis, gastric cancer (GC) accounts for the second leading cause of cancer-related death in the world [1]. A high level of evidence has demonstrated that preoperative or perioperative chemotherapy combined with gastrectomy could offer survival benefit to patients with GC as compared to surgical resection alone [2–4]. In addition to the improvement of survival rates, this multimodal strategy correlated with an increase in rates of curative gastrectomy and tumor-downstaging whilst not increasing the mortality and risks of postoperative complications [5]. Perioperative chemotherapy with surgery has been the standard therapeutic method for patients with gastric cancer classified as cT2 or higher in many Western countries [6].

Postoperative chemotherapy is recommended for patients who have received preoperative chemotherapy followed by curative surgery, regardless of the pathological status after surgery [7, 8]. However, no prospective randomized controlled trials (RCTs) that compared perioperative chemotherapy with preoperative chemotherapy alone have been published to date, the necessity of the component of perioperative treatment, chemotherapy implemented in the postoperative phase, is still controversial. There have been several retrospective studies investigating the survival benefit of postoperative chemotherapy after neoadjuvant chemotherapy and curative resection; however, the results were inconsistent and controversial [9–11]. Additionally, it remains inconclusive whether postoperative chemotherapy is necessary for patients with ypT1-2N0 gastric cancer who have a good response to preoperative chemotherapy or favorable pathological stage [6, 11]. The poor fitness after preoperative chemotherapy and surgery is one of the main reasons for the absence or incompleteness of postoperative chemotherapy [12]. Even in clinical trials, the planned postoperative chemotherapy was actually completed in less than 50% of patients, and the completion of postoperative chemotherapy is full of challenges [2, 11, 13]. In this context, evaluating the survival benefit and elucidating the necessity of postoperative chemotherapy has clinically meaningful benefit and would allow more well-founded decision about the implementation of the postoperative treatment in patients with gastric cancer, particularly in ypT1-2N0 patients for whom the oncological outcomes were more promising [14].

In the context of the limited data and the absence of prospective trails, this retrospective study aimed to investigate the impact of postoperative chemotherapy on the survival of patients with ypT1-2N0 gastric cancer, and analyzed whether the postoperative treatment was necessary for these patients.

## Methods

### Patient selection

We retrospectively reviewed the patients from our prospectively designed database who received curative gastrectomy with lymphadenectomy for gastric cancer between March 2016 and December 2020 at the department of general surgery of Peking Union Medical College Hospital. The inclusion criteria of our study were as follows: (1) histopathological evidence of gastric cancer examined by endoscopic biopsy; (2) locally advanced gastric cancer before preoperative chemotherapy and surgery (cT2-T4N0-3); (3) no distant metastasis; (4) received preoperative chemotherapy followed by radical gastrectomy with lymphadenectomy; (5) pathological classification of ypT1–2N0. The exclusion criteria were as follows: (1) received radical gastrectomy directly without preoperative chemotherapy; (2) received preoperative radiotherapy; (3) distant metastasis; (4) suffering from other malignancies; (5) suboptimal lymphadenectomy; (6) incomplete information on diagnosis and therapy.

### Patients’ characteristics

Demographic and clinicopathologic characteristics were grouped into categorical variables for analysis except for age, body mass index (BMI), tumor size and the number of lymph nodes. These covariates included gender (female and male), clinical T stage (cT2, cT3, cT4), clinical nodal status (cN− and cN+), neoadjuvant chemotherapy (NACT) regimen (SOX and XELOX), number of NACT cycles (2, 3, 4), tumor location (upper third, middle third and lower third), type of resection (subtotal and total), Lauren type (intestinal and diffuse/mixed), grades of differentiation (well/moderate and poor), signet ring cell (no and yes), pathological T stage (ypT1 and ypT2), lymphovascular invasion (no and yes) and pathological response (CAP 0, CAP 1, CAP 2, and CAP 3).

### Preoperative evaluation

The methods of evaluating clinical TNM stage based on preoperative endoscopic ultrasonography (EUS) and contrast-enhanced computed tomography (CT) completed with biopsy for histopathological diagnosis where appropriate, and expressed as cTNM according to the 8th edition American Joint Committee on Cancer (AJCC) Staging Manual. According to the guidelines of National Comprehensive Cancer Network (NCCN) and European Society for Medical Oncology (ESMO), patients with cT2 or cT2+ gastric cancer are recommended to receive preoperative chemotherapy regardless of lymph node status [15, 16]. Indication for preoperative chemotherapy was actually evaluated for each patient through a multidisciplinary tumor board, included surgeons, oncologists, radiologists, pathologists, and endoscopists.

### Preoperative and postoperative chemotherapy

The preoperative S-1 plus oxaliplatin (SOX) regimen consists of 130 mg/m^2^ oxaliplatin administered intravenously on day 1 and 80 mg/m^2^ S-1 administered orally once a day on days 1–14, while XELOX regimen consists of 130 mg/m^2^ oxaliplatin administered intravenously on day 1 and 1000 mg/m^2^ capecitabine (Xeloda) administered orally twice a day on days 1–14. The perioperative treatment was repeated two to four times every three weeks according to the clinical stages. Postoperative chemotherapy was completed in 56 patients (41.8%). The regimens were same as the preoperative regimens in most patients, and the total cycle of postoperative treatment was 4 to 6.

### Pathological evaluation after preoperative chemotherapy

Patients had a pathological stage (ypTNM) after the comprehensive review of two pathologists. The recommendations of College of American Pathologists (CAP) were adopted to assess the pathological response of gastrectomy specimens to preoperative chemotherapy, a four-category system was designated for grading tumor regression [17].

### Follow-up

Overall survival (OS) was calculated from the initiation of preoperative chemotherapy to death from any causes. Follow-up was performed through the telephone, the last follow-up was in June 2022. Date were censored if patients were alive at last follow-up evaluation.

### Statistical analysis

Categorical variables were described as frequency (percentage), continuous variables were described as mean (standard deviation). Differences between groups were analyzed by *χ*^2^ test or Fisher’s exact test and Student’s *t* test for categorical variables and continuous variables, respectively. Survival curves for OS were evaluated using the Kaplan-Meier method, and the log-rank test was used to compare survival difference. The Cox regression analysis was adopted to assess the prognostic risk of demographic and clinicopathologic characteristics on OS, and the statistically significant factors from the univariate analysis were then taken into the final multivariable analysis. Statistical analyses were performed using SPSS 22.0 (SPSS Inc., Chicago, IL, USA) and GraphPad Prism 8 (GraphPad Prism Software, Inc., San Diego, CA, USA), and *p* values < 0.05 was considered statistically significant.

## Results

### Patients’ characteristics

We identified 134 patients with gastric adenocarcinoma that met the inclusion criteria. Of these, 56 (41.8%) patients received postoperative chemotherapy after preoperative and surgical resection, 78 (58.2%) were treated with preoperative followed by surgical resection. Patients’ demographic and clinicopathologic characteristics are presented in Table 1. There were no statistically significant differences in terms of age, gender, BMI, number of resected lymph nodes, tumor size, preoperative chemotherapy regimen, cycle of preoperative chemotherapy between pCHT group and no pCHT group (all *p* > 0.05). Distributions of clinical T stage, clinical nodal status, tumor location, type of resection, Lauren type, differentiated degree, signet ring cell features, pathological T stage, lymphovascular invasion, and pathological response were also well balanced between the two groups (all *p* > 0.05).

**Table 1:** Demographic and clinicopathologic data of the patients with or without postoperative chemotherapy

Variable	All cohort	pCHT	no pCHT	***P*** value†
	***n*** = 134	***n*** = 56	***n*** = 78	
Age (years)				0.367‡
Mean (SD)	54.2 (10.8)	53.4 (11.5)	54.8 (10.2)	
Gender				0.519
Female	57 (42.5%)	22 (39.3%)	35 (44.9%)	
Male	77 (57.5%)	34 (60.7%)	43 (55.1%)	
BMI				0.672‡
Mean (SD)	21.0 (5.7)	21.4 (5.3)	20.7(6.2)	
Tumor size (cm)				0.245‡
Mean (SD)	3.9 (2.0)	4.1 (1.9)	3.8 (2.1)	
Clinical T stage				0.170
cT2	58 (43.3%)	19 (33.9%)	39 (50.0%)	
cT3	57 (42.5%)	27 (48.2%)	30 (38.5%)	
cT4	19 (14.2%)	10 (17.9%)	9 (11.5%)	
Clinical nodal status				0.601
cN−	70 (52.2%)	31 (55.4%)	39 (50.0%)	
cN+	64 (47.8%)	25 (44.6%)	39 (50.0%)	
NACT regimen				0.625
SOX	114 (85.1%)	49 (87.5%)	65 (83.3%)	
XELOX	20 (14.9%)	7 (12.5%)	13 (16.7%)	
No. of NACT cycles				0.381
2	15 (11.2%)	8 (14.3%)	7 (9.0%)	
3	36 (26.9%)	17 (30.4%)	19 (24.4%)	
4	83 (61.9%)	31 (55.3%)	52 (66.6%)	
Tumor location				0.860
Upper third	39 (29.1%)	16 (28.6%)	23 (29.5%)	
Middle third	44 (32.8%)	20 (35.7%)	24 (30.8%)	
Lower third	51 (38.1%)	20 (35.7%)	31 (39.7%)	
Type of resection				0.502
Subtotal	72 (53.7%)	32 (57.1%)	40 (51.3%)	
Total	62 (46.3%)	24 (42.9%)	38 (48.7%)	
Lauren type				0.138
Intestinal	86 (64.2%)	40 (71.4%)	46 (59.0%)	
Diffuse/mixed	48 (35.8%)	16 (28.6%)	32 (41.0%)	
Differentiation				0.934
Well/moderate	88 (65.7%)	37 (66.1%)	51 (65.4%)	
Poor	46 (34.3%)	19 (33.9%)	27 (34.6%)	
Signet ring cell				0.110
No	98 (73.1%)	45 (80.4%)	53 (67.9%)	
Yes	36 (26.9%)	11 (19.6%)	25 (32.1%)	
Pathological T stage				0.370
ypT1	51 (38.1%)	24 (42.9%)	27 (34.6%)	
ypT2	83 (61.9%)	32 (57.1%)	51 (65.4%)	
No. of lymph nodes				
Mean (SD)	29 (10)	28 (9)	30(12)	0.332‡
Lymphovascular invasion				0.153
No	80 (59.7%)	29 (51.8%)	51 (65.4%)	
Yes	54 (40.3%)	27 (48.2%)	27 (34.6%)	
Pathological response (CAP)				0.316
CAP 0	23 (17.2%)	12 (21.4%)	11 (14.1%)	
CAP 1	30 (22.4%)	11 (19.6%)	19 (24.4%)	
CAP 2	41 (30.6%)	20 (35.7%)	21 (26.9%)	
CAP 3	40 (29.8%)	13 (23.2%)	27 (34.6%)	

*pCHT p*ostoperative chemotherapy, *SD* standard deviation, *BMI* body mass index, *NACT* neoadjuvant chemotherapy, *yp p*athological status after neoadjuvant chemotherapy, *CAP* College of American Pathologists

†*χ*^2^ test, except

‡Student’s *t* test

### Impact of postoperative chemotherapy on overall survival

**Fig. 1:**
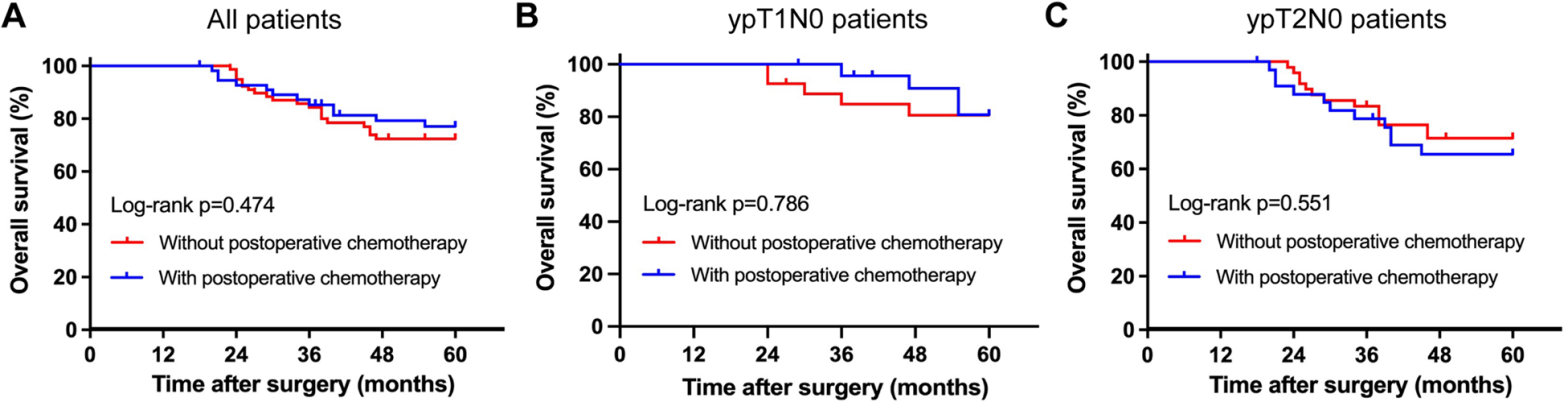
Overall survival (OS) based on whether patients received postoperative chemotherapy. **A** OS for all patients. **B** OS for ypT1N0 patients. **C** OS for ypT2N0 patients

The median follow-up for entire study was 53 months, no patient was lost during the follow-up. There was no significant difference in OS between pCHT group and no pCHT group (*p* = 0.474). The 5-year OS rate was 76.4% for the pCHT group and 72.6% for the no pCHT group (Fig. 1A). After stratification according to pathological T stage, postoperative chemotherapy also showed no benefit to the OS for ypT1N0 or ypT2N0 patients (79.6% vs. 80.0%, *p* = 0.786; 66.8% vs. 72.4%, *p* = 0.551) (Fig. 1B, C).

### Analysis of independent risk factors

Univariable Cox regression analysis identified the following several potential risk factors of OS: clinical T3–4 stage (cT3 vs. cT2: HR 3.073, 95% CI 0.991–9.532, *p* = 0.052; cT4 vs. cT2: HR 5.685, 95% CI 1.405–23.002, *p* = 0.015), clinical lymph node metastasis (HR 1.974, 95% CI 1.010–3.860, *p* = 0.047), poorly differentiated degree (HR 1.883, 95% CI 0.978–3.623, *p* = 0.058) and lymphovascular invasion (HR 1.770, 95% CI 0.920–3.403, *p* = 0.052). Stepwise selection of variables for multivariable Cox regression analysis identified that clinical T stage (cT3 vs. cT2: HR 2.875, 95% CI 0.998–8.281, *p* = 0.050; cT4 vs. cT2: HR 7.382, 95% CI 2.569–21.211, *p* < 0.001) as being independent risk factors associated with OS (Table 2).

**Table 2:** Univariable and multivariable Cox regression analysis of factors associated with overall survival in ypT1-2N0 patients

Variable	Univariable analysis		Multivariable analysis	
	HR (95% CI)	***P*** value	HR (95% CI)	***p*** value
Age (years)	1.017 (0.991–1.043)	0.193	–	
Gender			–	
Female	1 (ref.)			
Male	1.033 (0.533–2.004)	0.923		
BMI	1.057 (0.952–1.175)	0.297	–	
Tumor size (cm)	1.191 (0.909–1.560)	0.204		
Clinical T stage				
cT2	1 (ref.)		1 (ref.)	
cT3	3.073 (0.991–9.532)	*0.052*	2.875 (0.998–8.281)	*0.050*
cT4	5.685 (1.405–23.002)	*0.015*	7.382 (2.569–21.211)	*< 0.001*
Clinical nodal status				
cN−	1 (ref.)		1 (ref.)	
cN+	1.974 (1.010–3.860)	*0.047*	1.099 (0.430–2.807)	0.343
No. of NACT cycles			–	
2	1 (ref.)			
3	1.266 (0.456–3.516)	0.651		
4	0.574 (0.212–1.559)	0.276		
Tumor location			–	
Upper third	1 (ref.)			
Middle third	1.211 (0.560–2.619)	0.627		
Lower third	0.641 (0.277–1.484)	0.299		
Type of resection			–	
Subtotal	1 (ref.)			
Total	1.264 (0.658–2.429)	0.482		
Lauren type			–	
Intestinal	1 (ref.)			
Diffuse/mixed	1.405 (0.725–2.726)	0.314		
Differentiation				
Well/moderate	1 (ref.)		1 (ref.)	
Poor	1.883 (0.978–3.623)	0.058	1.399 (0.537–3.645)	0.492
Signet ring cell			–	
No	1 (ref.)			
Yes	1.674 (0.867–3.231)	0.125		
Pathological T stage			–	
ypT1	1 (ref.)			
ypT2	1.237 (0.619–2.474)	0.547		
No. of lymph nodes	1.031 (0.947–1.122)	0.487	–	
Lymphovascular invasion				
No	1 (ref.)		1 (ref.)	
Yes	1.770 (0.920–3.403)	0.052	1.029 (0.408–2.598)	0.251
Pathological response			–	
CAP 0	1 (ref.)			
CAP 1	1.701 (0.512–5.651)	0.386		
CAP 2	1.550 (0.494–4.870)	0.453		
CAP 3	2.352 (0.767–7.219)	0.135		
Postoperation chemotherapy				
No	1 (ref.)			
Yes	0.910 (0.473–1.750)	0.777		

*p* values < 0.05 are in italic

*HR* hazard ratio, *CI* confidence interval, *BMI* body mass index, *NACT* neoadjuvant chemotherapy, *yp* pathological status after neoadjuvant chemotherapy, *CAP* College of American Pathologists

### Stratification by risk factors

**Fig. 2:**
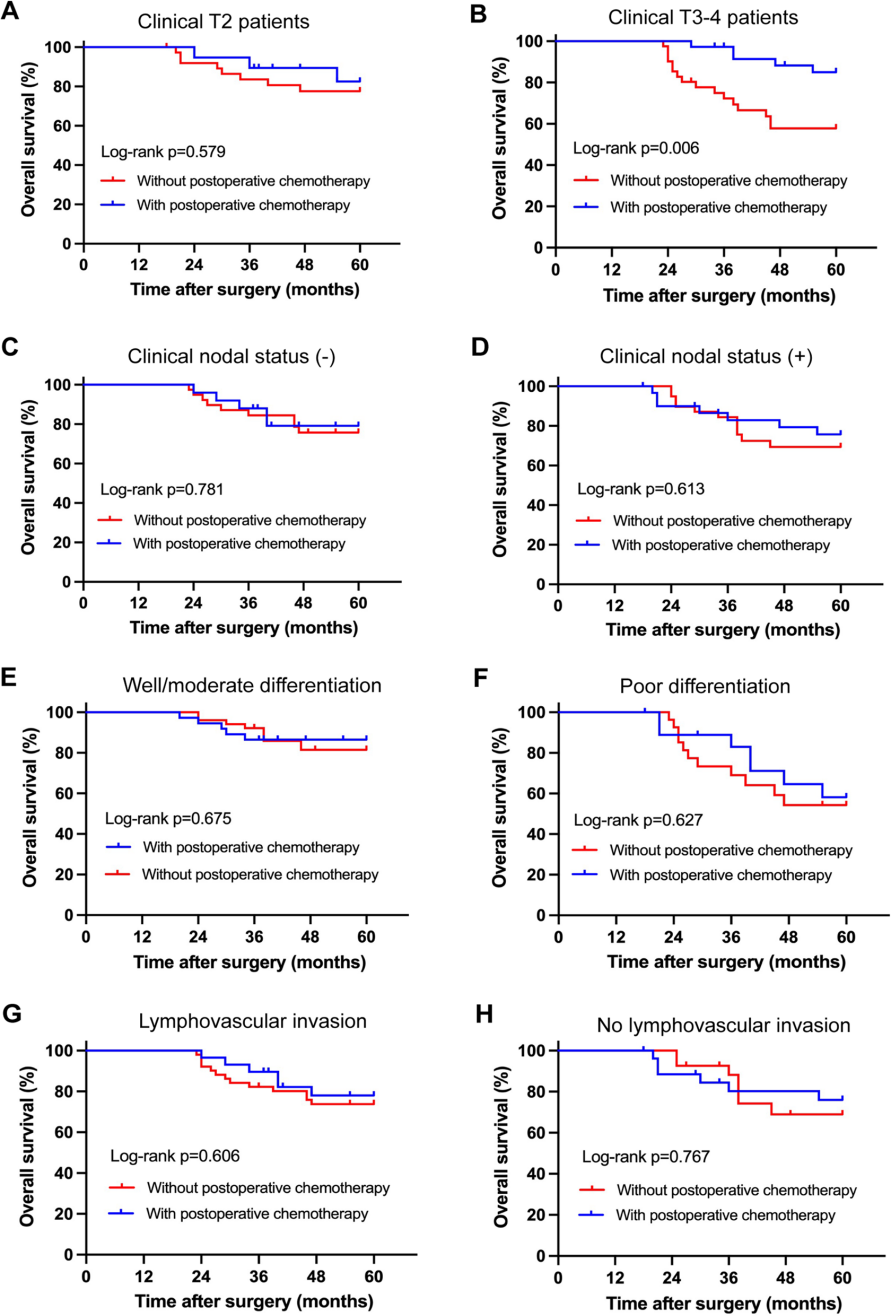
Overall survival (OS) based on whether patients received postoperative chemotherapy. **A** OS for clinical T2 patients. **B** OS for clinical T3–4 patients. **C** OS for patients without clinical lymph node metastasis. **D** OS for patients with clinical lymph node metastasis. **E** OS for patients with well/moderate differentiation. **F** OS for patients with poor differentiation. **G** OS for patients with lymphovascular invasion. **H** OS for patients without lymphovascular invasion

Among 76 patients whose clinical T stage was T3–4 before preoperative chemotherapy, 37 (48.7%) of them received postoperative chemotherapy. Kaplan–Meier survival analysis demonstrated that postoperative chemotherapy could bring benefit to the OS in these clinical T3–4 patients. The 5-year OS rate of these clinical T3–4 patients who received postoperative chemotherapy was 86.4%, significantly higher than 57.8% of those without postoperative chemotherapy (*p* = 0.006). However, postoperative chemotherapy brought no significant benefit to the OS for clinical T2 patients (82.6% vs. 78.2%, *p* = 0.579) (Fig. 2A, B). In addition, among patients with clinical lymph node metastasis, poorly differentiated degree or lymphovascular invasion, postoperative chemotherapy showed no survival benefits, as well as for patients without these factors (Fig. 2C–H). Moreover, postoperative chemotherapy and clinical T stage were further proved to be independent prognostic factors for clinical T3–4 patients (postoperative chemotherapy: HR 0.132, 95% CI 0.051–0.345, *p* < 0.001; clinical T stage: HR 3.908, 95% CI 1.039–14.705, *p* = 0.044) (Table 3).

**Table 3:** Univariable and multivariable Cox regression analysis of factors associated with overall survival in ypT1-2N0 patients whose clinical stage was T3–4 before preoperative chemotherapy

Variable	Univariable analysis		Multivariable analysis	
	HR (95% CI)	***P*** value	HR (95% CI)	***p*** value
Age (years)	1.210 (0.851-1.343)	0.176	–	
Gender			–	
Female	1 (ref.)			
Male	1.112 (0.505–2.449)	0.793		
BMI	1.126 (0.970–1.163)	0.108	–	
Tumor size (cm)	1.348 (0.986–2.169)	0.262	–	
Clinical T stage				
cT3	1 (ref.)		1 (ref.)	
cT4	2.740 (1.209–6.206)	*0.016*	3.908 (1.039–14.705)	*0.044*
Clinical nodal status			–	
cN−	1 (ref.)			
cN+	1.509 (0.685–3.324)	0.308		
No. of NACT cycles			–	
2	1 (ref.)			
3	1.348 (0.558–3.255)	0.506		
4	1.082 (0.362–3.229)	0.888		
Tumor location			–	
Upper third	1 (ref.)			
Middle third	2.143 (0.830–5.530)	0.115		
Lower third	1.252 (0.439–3.569)	0.675		
Type of resection			–	
Subtotal	1 (ref.)			
Total	1.388 (0.630–3.058)	0.416		
Lauren type			–	
Intestinal	1 (ref.)			
Diffuse/mixed	1.068 (0.485–2.352)	0.871		
Differentiation				
Well/moderate	1 (ref.)		1 (ref.)	
Poor	3.205 (1.278–8.036)	*0.013*	2.089 (0.580–7.527)	0.260
Signet ring cell				
No	1 (ref.)		1 (ref.)	
Yes	2.693 (1.161–6.249)	*0.021*	0.969 (0.193–3.059)	0.709
Pathological T stage			–	
ypT1	1 (ref.)			
ypT2	1.279 (0.574–2.847)	0.547		
No. of lymph nodes	0.957 (0.846–1.302)	0.425	–	
Lymphovascular invasion			–	
No	1 (ref.)			
Yes	1.509 (0.685–3.326)	0.307		
Pathological response			–	
CAP 0	1 (ref.)		1 (ref.)	
CAP 1	1.393 (0.408–4.760)	0.597	0.678 (0.180–2.546)	0.565
CAP 2	1.647 (0.442–6.136)	0.457	2.009 (0.488–8.270)	0.334
CAP 3	3.605 (1.109–11.714)	0.033	2.112 (0.518–8.611)	0.297
Postoperation chemotherapy				
No	1 (ref.)		1 (ref.)	
Yes	0.242 (0.101–0.584)	*0.002*	0.132 (0.051–0.345)	*< 0.001*

*p* values < 0.05 are in italic

*HR* hazard ratio, *CI* confidence interval, *BMI* body mass index, *NACT* neoadjuvant chemotherapy, *yp* pathological status after neoadjuvant chemotherapy, *CAP* College of American Pathologists

## Discussion

Perioperative chemotherapy has been evaluated in improving the survival of patients with gastric cancer over the last few decades [18]. The famous MAGIC trial, FNLCLCC/FFCD trial and FLOT4 trial have gradually established perioperative chemotherapy to be an effective strategy for resectable gastric cancer [2, 3, 19]. Subsequent studies further provided supporting data for the survival benefit of the multimodal treatment and aimed at optimizing chemotherapy scheme [20–22]. However, the comparatively poor fitness of patients who have already received the debilitating preoperative chemotherapy in combination with surgery resulted in fewer than 50% of patients completing the postoperative chemotherapy according to the protocol in above studies, and there have been no data from prospective randomized clinical trials evaluating the survival benefit of continued perioperative chemotherapy postoperatively. Several retrospective studies [9–11, 23] have revealed conflicting outcomes regarding the necessity of continued perioperative chemotherapy postoperatively and left uncertainty as to whether these patients should be targeted for postoperative chemotherapy. Drawing definite conclusions for the whole cohort from the published retrospective analyses is full of challenges.

Adjuvant chemotherapy is recommended in gastric cancer patients who have received upfront radical gastrectomy and have pT3-4 lesions or lymph node metastasis, while patients with pT1-2N0 stage are not recommended to receive adjuvant chemotherapy in many guidelines [8, 16, 24–26]. Besides, these guidelines or studies did not elucidate whether postoperative chemotherapy should be administered in gastric cancer patients with ypT1-2N0 stage after preoperative chemotherapy. Stage ypT1-2N0 gastric cancer was considered as stage I disease according to a post-neoadjuvant therapy staging system proposed by the American Joint Committee on Cancer (AJCC) [27]. Patients with stage ypT1-2N0 gastric cancer have either initially favorable pathological stage or good response to preoperative therapy that may obviate the necessity of postoperative chemotherapy. However, we observed that tumors still grew back subsequently after the treatment in some patients with stage ypT1-2N0 gastric cancer in clinical practice, so we focused on the specific gastric cancer patient subgroup whose pathological stage was ypT1-2N0 after preoperative chemotherapy and radical gastrectomy at present study.

Among the whole cohort, the present study revealed that patients who received perioperative chemotherapy postoperatively had no survival benefit, compared with patients undergoing preoperative chemotherapy alone, and the 5-year OS rate was 76.4% and 72.6% respectively for patients with and without postoperative chemotherapy (*p* = 0.474). Subgroup analyses also demonstrated that postoperative chemotherapy had no survival benefit in the 5-year OS rates of patients with stage ypT1N0 or ypT2N0 gastric cancer (*p* = 0.786; *p* = 0.551). Although adjuvant chemotherapy is not recommended for gastric cancer patients with pT1-2N0 stage in many guidelines, there have been studies drawing conclusions that postoperative chemotherapy could offer survival benefits to these pT2N0 patients with risk factors, such as larger tumor diameter, lymphovascular invasion, suboptimal lymphadenectomy, and poor differentiation [28–30]. Herein, we were inspired by these findings and speculated that whether postoperative chemotherapy could offer survival benefit to stage ypT1-2N0 gastric cancer patients who had risk factors. Multivariable analysis revealed that clinical T stage independently influenced prognosis (cT3 vs. cT2: HR 2.875, *p* = 0.050; cT4 vs. cT2: HR 7.382, *p* < 0.001), so clinical T3–4 stage could be perceived reasonably as the independent risk factor for ypT1-2N0 gastric cancer. Does the subgroup with risk factor benefit from postoperative chemotherapy? We further explored that there was an overall survival benefit for postoperative chemotherapy in clinical T3–4 patients, the 5-year OS rate of these patients who received postoperative chemotherapy was 86.4%, significantly higher than 57.8% of those without postoperative chemotherapy (*p* = 0.006). No survival benefit for postoperative chemotherapy was identified in clinical T2 patients. Moreover, postoperative chemotherapy was proved to be an independently positive prognostic factor for clinical T3–4 patients (HR 0.132, *p* < 0.001).

We acknowledge that the present study contains certain limitations. Due to its retrospective nature and relatively limited number of patients at a single institution, potential selection bias and excessive hazard ratios in the stratified analysis might exist. Only SOX and XELOX regimen was involved in our study, adopting other schemes (i.e., FLOT) might have different effect on the results. The available survival information is only the overall survival at present study, in addition, the follow-up period is not long enough, which might hide the significance of some factors in survival to a certain extent. Despite the limitations above, the present study supports the conclusion that postoperative chemotherapy could provide OS benefits for the selected group of patients. Furthermore, prospective randomized clinical trials are required to prove the necessity of perioperative chemotherapy postoperatively for gastric cancer patients, including ypT1-2N0 gastric cancer patients.

## Conclusion

To the best of our knowledge, this is the first study evaluating the impact of postoperative chemotherapy on patients with ypT1-2N0 gastric cancer. This retrospective study demonstrated that the postoperative component of perioperative chemotherapy might have clinically meaningful benefit for patients with ypT1-2N0 gastric cancer whose clinical T stage was T3–4 before preoperative chemotherapy, and postoperative chemotherapy was an independently positive prognostic factor for these patients. Our findings are expected to be supported by future prospective studies.

## Data Availability

The datasets generated during and/or analyzed during the current study are available from the corresponding author on reasonable request.

## Abbreviations

GC: Gastric cancer
OS: Overall survival
HR: Hazard ratio
CI: Confidence interval
RCTs: Randomized controlled trials
BMI: Body mass index
NACT: Neoadjuvant chemotherapy
TNM: Tumor-node-metastasis
CAP: College of American Pathologists
yp: Pathological status after neoadjuvant chemotherapy
SD: Standard deviation
